# Supplementary figures and images for: Metabarcoding monitoring analysis: the pros and cons of using co-extracted environmental DNA and RNA data to assess offshore oil production impacts on benthic communities
Source: PeerJ. 2017 May 17;5:e3347. doi: 10.7717/peerj.3347 (PMC5437860; doi:10.7717/peerj.3347)

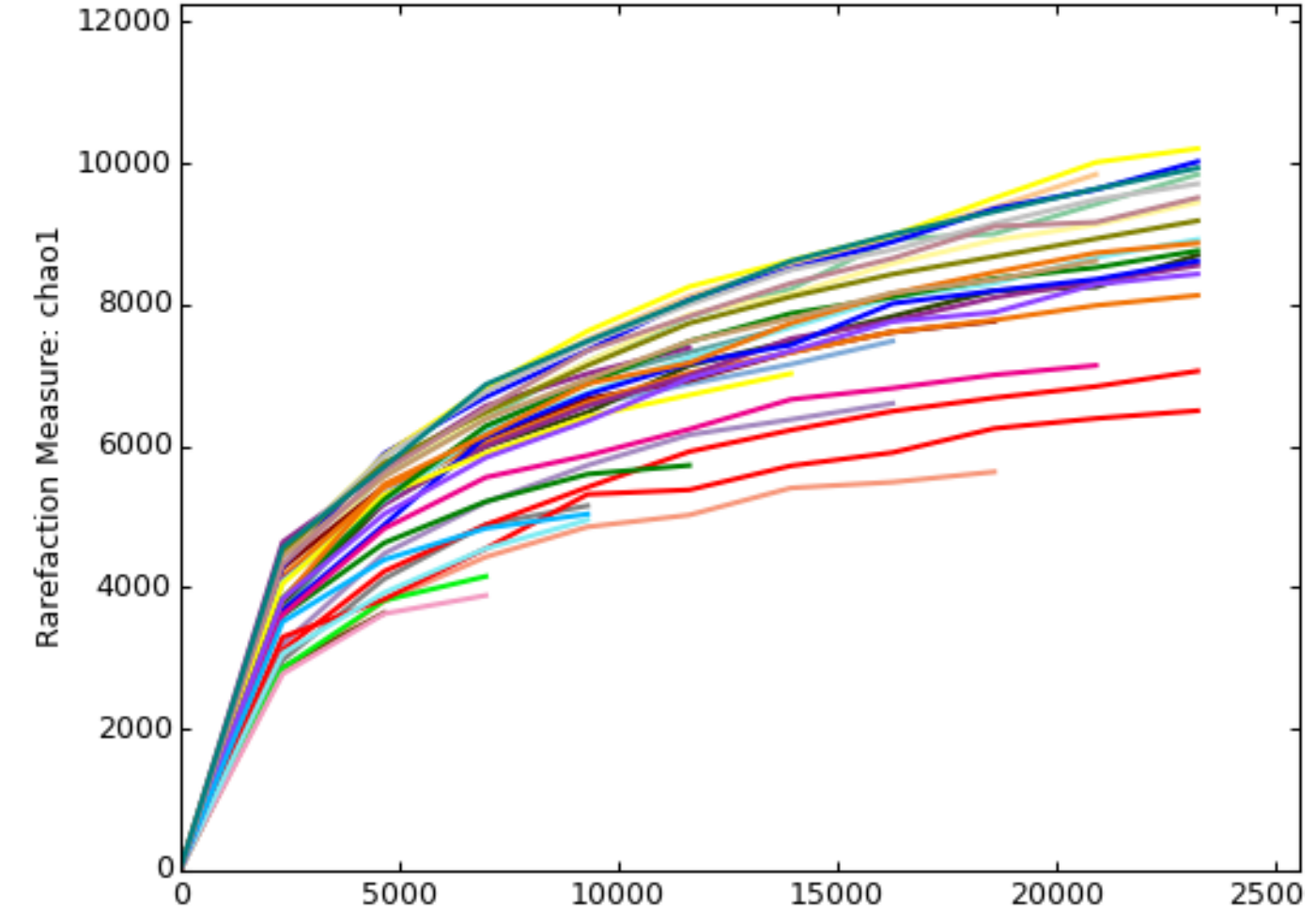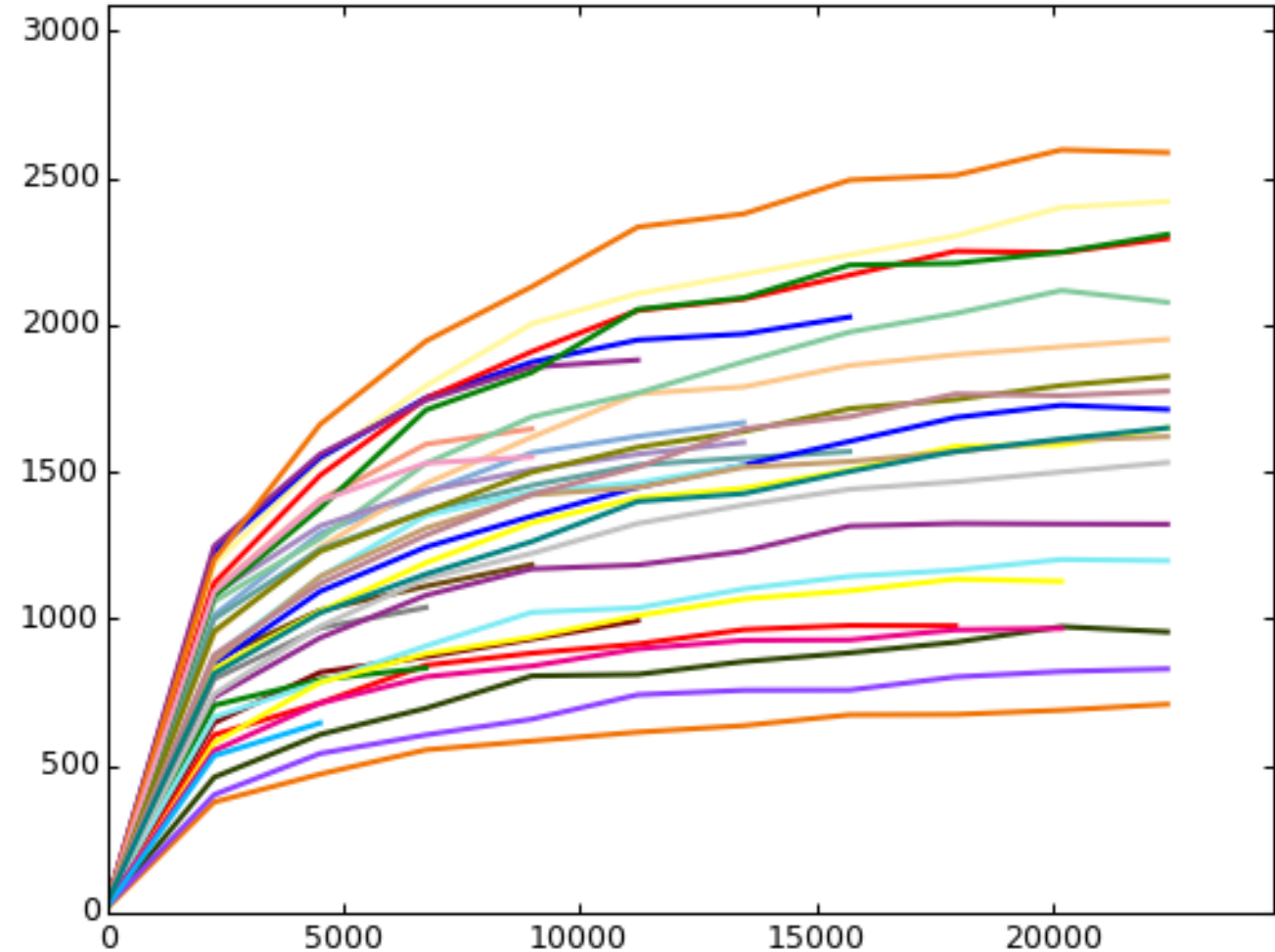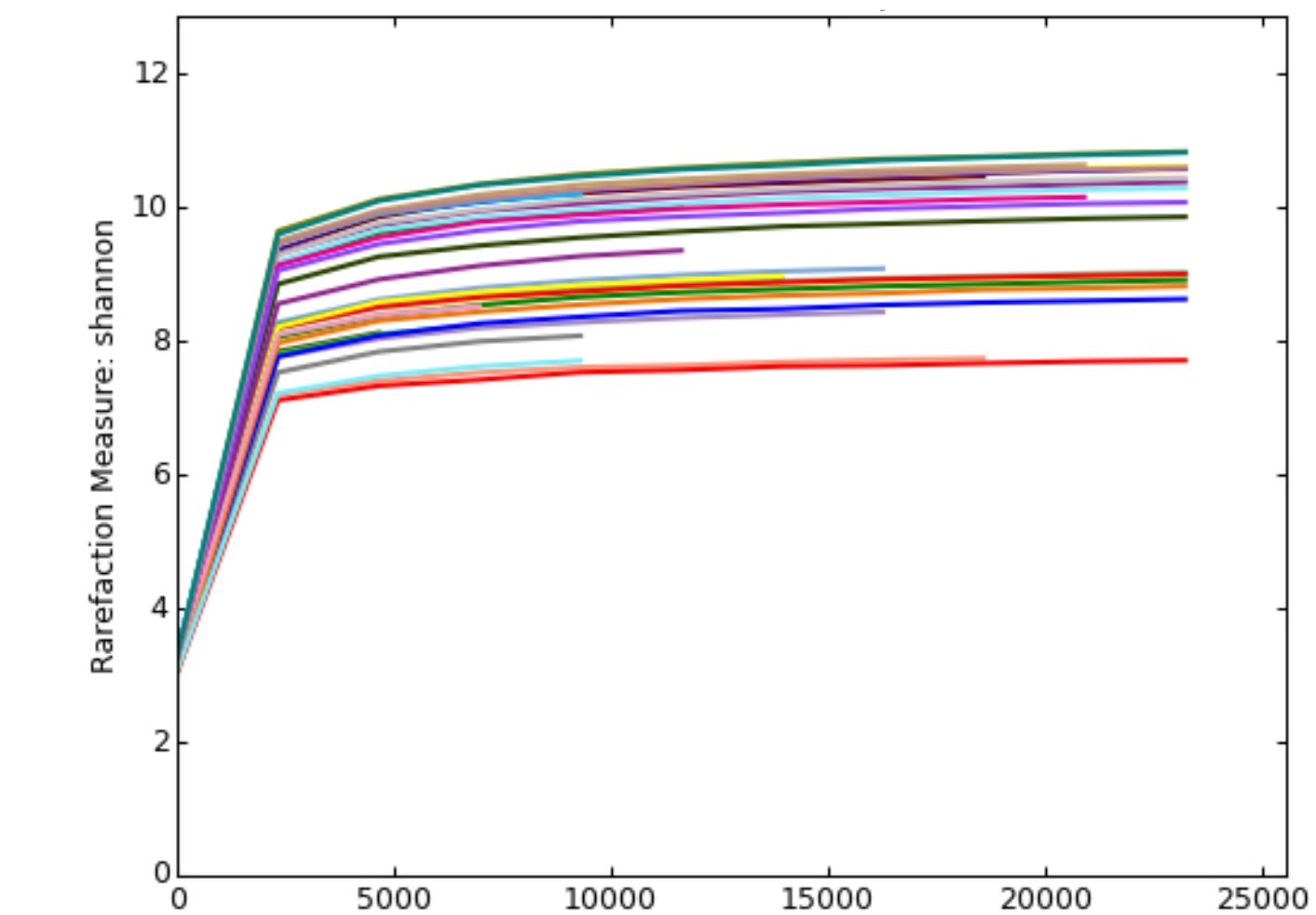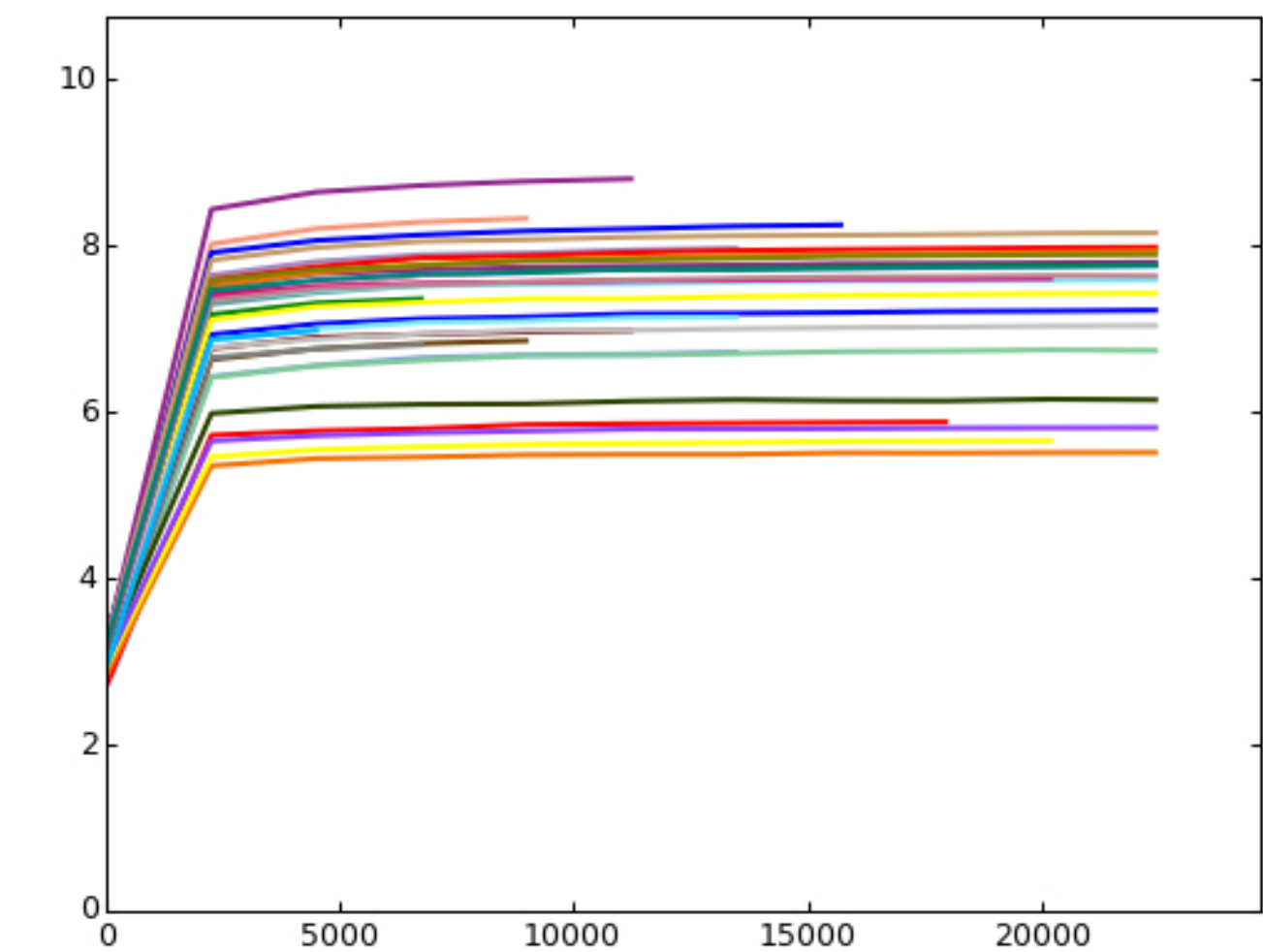

A) Bacteria

B) Eukaryotes

Supplement: Figure S11 [file peerj-05-3347-s001.pdf]
