# Supplementary material for: Metabarcoding monitoring analysis: the pros and cons of using co-extracted environmental DNA and RNA data to assess offshore oil production impacts on benthic communities
Source: PeerJ. 2017 May 17;5:e3347. doi: 10.7717/peerj.3347 (PMC5437860; doi:10.7717/peerj.3347)
Supplement: Table S2 [file peerj-05-3347-s003.docx]

**Table S2**. Analytical methods used for the laboratory analysis of the physico-chemical characterization of sediments. Adapted from Johnston et al. (2014) and Skilton et al. (2015).

| **Analyte** | **Method number** | **Method description** | **Detection limit** |
| --- | --- | --- | --- |
| Organic content (AFDW) | APHA 2540 G 22nd ed. 2012. | Ignition in muffle furnace 550 °C, 6 hr, gravimetric. | 0.04 g/100 g dry wt 0.010 |
| Heavy metals, trace As,Cd,Cr,Cu,Ni,Pb,Zn,Hg | PSP 2002 mod./APHA metals by ICP-MS | Dried sample, <2 mm fraction. Nitric/Hydrochloric acid digestion, ICP-MS, trace level | 0.010 - 0.4 mg/kg dry w |
| Total Recoverable Barium | PSP 2002 mod./APHA metals by ICP-MS | Dried sample, sieved as specified (if required). 1, 5, 9 Nitric/Hydrochloric acid digestion, ICP-MS, trace level. US EPA 200.2. | 0.02 mg/kg dry wt 40 |
| Total Recoverable Iron Total | PSP 2002 mod./APHA metals by ICP-MS | Dried sample, sieved as specified (if required). 1, 5, 9 Nitric/Hydrochloric acid digestion, ICP-MS, screen level. USEPA 200.2 | 40 mg/kg dry wt 1.0 |
| Total Recoverable Manganese | PSP 2002 mod./APHA metals by ICP-MS | Dried sample, sieved as specified (if required). 1, 5, 9 Nitric/Hydrochloric acid digestion, ICP-MS, screen level. USEPA 200.2 | 1.0 mg/kg dry wt |
| Polycyclic Aromatic Hydrocarbons | ESEPA 8270C | Sonication extraction, SPE (solid phase extraction) cleanup, GC-MS SIM (gas chromatography-mass spectroscopy selective ion monitoring) analysis. | 0.002 - 0.010 mg/kg dry wt |
| Total Petroleum Hydrocarbons | US EPA 8015B/MfE Petroleum Industry Guidelines. | Sonication extraction, Silica cleanup, GC-FID (flame ionising detection) analysis . | 8 - 70 mg/kg dry wt |
