# Supplementary material for: Metabarcoding monitoring analysis: the pros and cons of using co-extracted environmental DNA and RNA data to assess offshore oil production impacts on benthic communities
Source: PeerJ. 2017 May 17;5:e3347. doi: 10.7717/peerj.3347 (PMC5437860; doi:10.7717/peerj.3347)
Supplement: Table S7 [file peerj-05-3347-s008.docx]

**Table S7:** Number of indicator operational taxonomic units (OTUs) per dataset associated with the near-field or far-field station groups.

| **Datasets** | | | **Non-parametric t-test with 999 permutations** |
| --- | --- | --- | --- |
|  |  |  | Indicator OTUs^1^ |
| *Bacteria* | *Trimmed by singletons* | *eDNA* | 179 |
|  |  | *eRNA* | 96 |
|  | *Trimmed by shared OTUs* | *eDNA* | 127 (-29%) |
|  |  | *eRNA* | 70 (-27%) |
| *Eukaryote* | *Trimmed by singletons* | *eDNA* | 128 |
|  |  | *eRNA* | 112 |
|  | *Trimmed by shared OTUs* | *eDNA* | 29 (-77%) |
|  |  | *eRNA* | 26 (-77%) |

^1^ Differences in percentage of indicator OTUs when compared to the trimmed by singletons dataset are displayed inside brackets.
