# Supplementary material for: Metabarcoding monitoring analysis: the pros and cons of using co-extracted environmental DNA and RNA data to assess offshore oil production impacts on benthic communities
Source: PeerJ. 2017 May 17;5:e3347. doi: 10.7717/peerj.3347 (PMC5437860; doi:10.7717/peerj.3347)
Supplement: Table S8 [file peerj-05-3347-s009.docx]

**Table S8:** Distance-based test for homogeneity of multivariate dispersions (Permdisp) analysis with 999 permutations, assessing the beta-diversity variance within the near-field and far-field station groups. Deviations are from centroids.

| **Datasets** | | | **Permdisp** | |
| --- | --- | --- | --- | --- |
|  |  |  | F-value | P-value |
| *Bacteria* | *Trimmed by singletons* | *eDNA* | 3.73 | 0.07 |
|  |  | *eRNA* | 0.04 | 0.83 |
|  | *Trimmed by shared OTUs* | *eDNA* | 1.13 | 0.30 |
|  |  | *eRNA* | 0.39 | 0.54 |
| *Eukaryotes* | *Trimmed by singletons* | *eDNA* | <0.01 | 0.95 |
|  |  | *eRNA* | 1.19 | 0.33 |
|  | *Trimmed by shared OTUs* | *eDNA* | 0.02 | 0.88 |
|  |  | *eRNA* | 0.03 | 0.86 |
