# Supplementary material for: Metabarcoding monitoring analysis: the pros and cons of using co-extracted environmental DNA and RNA data to assess offshore oil production impacts on benthic communities
Source: PeerJ. 2017 May 17;5:e3347. doi: 10.7717/peerj.3347 (PMC5437860; doi:10.7717/peerj.3347)
Supplement: Table S9 — Deviations are from centroids. Significant p-values are in bold. [file peerj-05-3347-s010.docx]

**Table S9:** Distance-based test for homogeneity of multivariate dispersions (Permdisp) analysis assessing the beta-diversity variance between biological replicates. Deviations are from centroids. Significant p-values are in bold.

| **Datasets** | | | **Permdisp** | |
| --- | --- | --- | --- | --- |
|  |  |  | F-value | P-value |
| *Bacteria* | *Trimmed by singletons* | *eDNA* | 5.05 | **0.03** |
|  |  | *eRNA* | 6.64 | **0.04** |
|  | *Trimmed by shared OTUs* | *eDNA* | 5.28 | **0.03** |
|  |  | *eRNA* | 3.87 | 0.09 |
| *Eukaryotes* | *Trimmed by singletons* | *eDNA* | 3.06 | 0.19 |
|  |  | *eRNA* | 3.47 | 0.21 |
|  | *Trimmed by shared OTUs* | *eDNA* | 2.22 | 0.27 |
|  |  | *eRNA* | 2.65 | 0.24 |
